# Critical appraisal of methodological quality and completeness of reporting in Chinese social science systematic reviews with meta‐analysis: A systematic review

**DOI:** 10.1002/cl2.70014

**Published:** 2025-01-19

**Authors:** Liping Guo, Sarah Miller, Wenjie Zhou, Zhipeng Wei, Junjie Ren, Xinyu Huang, Xin Xing, Howard White, Kehu Yang

**Affiliations:** ^1^ School of Basic Medical Sciences, Evidence‐Based Medicine Centre Lanzhou University Lanzhou China; ^2^ School of Public Health, Center for Evidence‐Based Social Science Lanzhou University Lanzhou China; ^3^ Innovation Laboratory of Evidence‐Based Social Science Lanzhou University Lanzhou China; ^4^ Campbell UK & Ireland, School of Social Sciences, Education and Social Work Queen's University Belfast Belfast UK; ^5^ School of Information Resource Management Renmin University Beijing China; ^6^ Research and Evaluation Centre London UK

**Keywords:** meta‐analysis, reporting completeness, social science, systematic reviews

## Abstract

**Background:**

A systematic review is a type of literature review that uses rigorous methods to synthesize evidence from multiple studies on a specific topic. It is widely used in academia, including medical and social science research. Social science is an academic discipline that focuses on human behaviour and society. However, consensus regarding the standards and criteria for conducting and reporting systematic reviews in social science is lacking. Previous studies have found that the quality of systematic reviews in social science varies depending on the topic, database, and country.

**Objectives:**

This study evaluates the completeness of reporting and methodological quality of intervention and non‐intervention systematic reviews in social science in China. Additionally, we explore factors that may influence quality.

**Search Methods:**

We searched three major Chinese electronic databases—CNKI, VIP, and Wangfang—for intervention and non‐intervention reviews in social science published in Chinese journals from 1 January 2009 to 2 December 2022.

**Selection Criteria:**

We included intervention and non‐intervention reviews; however, we excluded overviews, qualitative syntheses, integrative reviews, rapid reviews, and evidence syntheses/summaries. We also excluded meta‐analyses that used advanced methods (e.g., cross‐sectional, cumulative, Bayesian, structural equation, or network meta‐analyses) or that focused on instrument validation.

**Data Collection and Analysis:**

We extracted data using a coding form with publication information and study content characteristics. This study conducted pilot extraction and quality assessment with four authors and formal extraction and assessment with two groups of four authors each. PRISMA2020 and MOOSE were used to evaluate the reporting completeness of intervention and non‐intervention reviews. AMSTAR‐2 and DART tools were adopted to assess their methodological quality. We described the characteristics of the included reviews with frequencies and percentages. We used SPSS (version 26.0) to conduct a linear regression analysis and ANOVA to explore the factors that may influence both completeness of reporting and methodological quality.

**Main Results:**

We included 1176 systematic reviews with meta‐analyses published in Chinese journals between 2009 and 2022. The top three fields of publication were psychology (417, 35.5%), education (388, 33.0%), and management science (264, 22.4%). Four hundred and thirty‐two intervention reviews were included. The overall completeness of reporting in PRISMA and compliance rate of the methodological process in AMSTAT‐2 were 49.9% and 45.5%, respectively. Intervention reviews published in Chinese Social Science Citation Index (CSSCI) journals had lower reporting completeness than those published in non‐CSSCI journals (46.7% vs. 51.1%), similar to methodological quality (39.6% vs. 47.9%). A few reviews reported the details on registration (0.2%), rationality of study selection criteria (1.6%), sources of funding for primary studies (0.2%), reporting bias assessment (2.8%), certainty of evidence assessment (1.2%), and sensitivity analysis (107, 24.8%). Seven hundred and forty‐four non‐intervention reviews were included. The overall completeness of reporting in MOOSE and compliance rate of the methodological process in DART were 51.8% and 50.5%, respectively. Non‐intervention reviews published in CSSCI journals had higher reporting completeness than those published in non‐CSSCI journals (53.3% vs. 50.3%); however, there was no difference in methodological quality (51.0% vs. 50.0%). Most reviews did not report the process and results of selection (80.8%), and 58.9% of reviews did not describe the process of data extraction; only 9.5% assessed the quality of included studies; while none of the reviews examined bias by confounding, outcome reporting bias, and loss to follow‐up. An improving trend over time was observed for both intervention and non‐intervention reviews in completeness of reporting and methodological quality (PRISMA: *β* = 0.24, *p* < 0.01; AMSTAR‐2: *β* = 0.17, *p* < 0.01; MOOSE: *β* = 0.34, *p* < 0.01; DART: *β* = 0.30, *p* < 0.01). The number of authors and financial support also have a positive effect on quality.

**Authors' Conclusions:**

Completeness of reporting and methodological quality were low in both intervention and non‐intervention reviews in Chinese social sciences, especially regarding registration, protocol, risk of bias assessment, and data and code sharing. The sources of literature, number of authors, publication year, and funding source declarations were identified as factors that may influence the quality of reviews. More rigorous standards and guidelines for conducting and reporting reviews are required in social science research as well as more support and incentives for reviewers to adhere to them.

## 1 PLAIN LANGUAGE SUMMARY

### 1.1 The methodological quality and reporting transparency in systematic reviews with meta‐analysis in Chinese social science need to be improved

The methodological quality and reporting completeness in systematic reviews with meta‐analysis in Chinese social science are currently low. But they show a significant trend of improvement, especially in non‐intervention reviews since 2014.

### 1.2 What is this review about?

High‐quality reviews and meta‐analyses are useful for collecting high‐quality evidence for policy makers. However, incomplete report of evidence could mislead investment into ineffective projects with an incomplete evidence. Besides, different methods could result in different conclusions on the same topic, potentially causing confusion for future researchers. This review evaluates the quality of methods and transparency of method used by systematic reviews in social sciences, and investigates their trends over time and influence factors. We include systematic reviews with meta‐analysis published in Chinese journals.

### 1.3 What is the aim of this review?

The review aims to evaluate the completeness of reporting of the method, and quality of these method of Chinese social science systematic reviews with meta‐analysis, and investigate the trend and factors that may influence their quality and reporting transparency.

### 1.4 What studies are included?

This study included 1048 reviews from Chinese journals, including 412 intervention and 626 non‐interventional reviews. These reviews were published between 2009 and 2022.

### 1.5 What are the main findings of this review?

This review indicated that the overall completeness of reporting and methodological quality in both intervention and non‐intervention reviews were low, but have shown improvement. particularly in non‐interventional reviews since 2014. Common weaknesses identified include a lack of registration, protocol, risk of bias assessment, and data and code sharing. Factors such as the sources of literature, number of authors, publication year, and funding source declaration also influenced the quality of the reviews.

The existing guidelines for the conduct and reporting of reviews primarily concerns reviews for the health sector. Researchers in social science should adapt healthcare reporting guidelines for systematic reviews and meta‐analyses so they are applicable to the social sciences. International organizations could publish these guidelines in multiple languages to promote global research. Research funders and journals may also consider requesting evidence producers adhere to these guidelines.

### 1.6 How up‐to‐date is this review?

The review authors conducted searches for studies up to December 2, 2022.

## 2 BACKGROUND

The practice of using evidence to inform decision making has grown in the social sciences field in China, with more systematic reviews and meta‐analyses being conducted. Systematic reviews aim to provide a comprehensive summary of the best available research on a specific question by synthesizing the results from multiple studies. These reviews used transparent procedures to search, evaluate, and synthesize research while minimizing bias. Owing to their integrative approach, systematic reviews are considered a more valuable reference for policy‐making and practice than individual studies. Consequently, the Oxford Centre of Evidence‐Based Medicine recommends systematic reviews/meta‐analyses as Level A evidence (level 1 evidence) in the evidence classification (Gates & March, 2016). The What Works movement in the United Kingdom uses systematic reviews to inform evidence‐based decision making (Gough et al., 2018).

High‐quality systematic reviews and meta‐analyses are effective sources of scientific evidence for decision makers and researchers to quickly understand the current progress of a research problem (Oliver & Dickson, 2016). The completeness of a systematic review's reporting depends on how well the research report is presented and the rigour of its methodology. Additionally, differences in methodology can result in contradictory conclusions from systematic reviews of the same topic, potentially misleading future researchers and decision makers (de Vrieze, 2018; Schalken & Rietbergen, 2017).

Mulrow (1987) evaluated 50 reviews published between June 1985 and June 1986 in 4 major medical journals: *Annals of Internal Medicine*, *Archives of Internal Medicine*, *Journal of the American Medical Association*, and *New England Journal of Medicine*. The study found that none of the reviews met all eight clear scientific criteria for assessing the quality of the included studies (Mulrow, 1987). Sacks et al. (1987) evaluated the adequacy of reporting in 83 English‐language meta‐analyses of randomized controlled trials in the medical field. The meta‐analyses were published between January 1966 and October 1986 and included six areas: study design, combinability, control of bias, statistical analysis, sensitivity analysis, and application of results. The study revealed that the completeness of reporting in the meta‐analyses was poor, with only 1–14 items out of 83 being fully reported (Sacks et al., 1987). After 10 years, Sacks et al. (1996) updated the study and found that the situation had not improved significantly (Sacks et al., 1996).

To improve the quality of meta‐analyses, Moher et al. (1999) issued the Quality of Reporting of Meta‐Analysis (QUOROM) guideline, focusing on the quality of meta‐analysis of randomized controlled trials (Moher et al., 1999). These guidelines were revised in 2009 and renamed the Preferred Reporting Items for Systematic Reviews and Meta‐Analysis (PRISMA), which also focuses on the completeness of reporting of systematic reviews. With advances in systematic review methodology and terminology, Page et al. (2021) developed the PRISMA 2020 statement for reporting systematic reviews with suggested modifications to the PRISMA 2009 statement published in 2021 (Page et al., 2021). Meanwhile, as the number of published meta‐analyses on observational studies in health increased, Stroup et al. (2000) proposed a reporting checklist for Meta‐analysis of Observational Studies in Epidemiology (MOOSE) to examine the reporting of meta‐analyses of observational studies. (Stroup et al., 2000).

In addition to the reporting checklist, Shea et al. (2009) developed a measurement tool to assess systematic reviews (AMSTAR) to evaluate the methodological quality of systematic reviews of randomized controlled trials (Shea et al., 2009). After receiving comments and feedback, the AMSTAR group revised AMSTAR and released AMSTAR‐2 in September 2017 (Shea et al., 2017), which also included non‐randomized studies of interventions (NRSI). Diekemper et al. (2015) developed a document and appraisal review tool (DART) for systematic reviews that explicitly included quality reviews for biases specific to observational studies (Diekemper et al., 2015).

Since the release of these guidelines, various studies have investigated the methodology and reporting quality of systematic reviews and meta‐analyses in medical research. Studies have been conducted in areas such as substance abuse (Kim et al., 2021), paediatrics (Shim et al., 2020), nursing (Jin et al., 2014), and orthopaedics (Gagnier & Kellam, 2013).

Recently, the number of systematic reviews and meta‐analyses in the social sciences has increased. Social science refers to any academic study or science that deals with the social and cultural aspects of human behaviour, focusing on the scientific study of human society and social relationships. Some researchers have evaluated the quality of systematic reviews in the social sciences. For instance, Kogut et al. (2019) assessed the reporting completeness of 40 systematic reviews of mathematics education and found shortcomings in search processes and reporting search methods (Kogut et al., 2019). Wang et al. (2021) examined the reporting quality of 96 Campbell Systematic Reviews and found that less than half (42%) were of high quality; however, the quality has improved since PRISMA statement, MOOSE checklist, and AMSTAR tools were introduced (Wang et al., 2021).

Chinese researchers have been increasingly conducting systematic reviews. To ensure their quality, they have introduced critical appraisal methods along with Chinese versions of critical appraisal tools (Ge et al., 2017; Moher et al., 2009; Tao et al., 2018; Tian et al., 2015; Xiong & Chen, 2011; Zhan, 2010; Zhang et al., 2015). Tian et al. (2017) compared the methodological quality and reporting completeness of 100 systematic reviews by authors from China and those from the United States and found them to be of similar quality (Tian et al., 2017). However, Bai et al. (2022) suggested that the reporting completeness of systematic reviews in the social sciences field was considered as critical low (Bai et al., 2022). It is worth noting that the data source used by them was limited to the CSSCI database, which only covered 500 of 2700 Chinese academic journals in the social sciences; thus, it is reasonable to suspect possible selection bias in their review. Therefore, the present study aimed to evaluate the completeness of reporting and review the methodological quality of systematic reviews in the social sciences in China and to assess the applicability of these tools in the Chinese context using content analysis.

## 3 OBJECTIVES

The present review includes three main objectives:

1. To assess the reporting completeness of intervention and non‐intervention systematic reviews published in Chinese social science journals based on the analyses of PRISMA and MOOSE guidelines.
2. To review the methodological quality of intervention and non‐intervention systematic reviews published in Chinese social science journals using the AMSTAR‐2 and DART standards.
3. To analyze the potential factors that may influence quality of intervention and non‐intervention systematic reviews published in Chinese social science journals using regression analysis.

## 4 METHODS

### 4.1 Protocol of the systematic review

The protocol was published in Campbell Systematic Reviews on 29 September 2022 (Guo et al., 2022).

### 4.2 Criteria for considering studies for this review

We included completed intervention and non‐intervention systematic reviews with meta‐analyses in the social sciences field published in Chinese journals between January 2009 and January 2022. If a review had been updated, the most recent version was selected for inclusion. Overviews of systematic reviews, qualitative evidence syntheses, integrative reviews, rapid reviews, and evidence syntheses or summaries were excluded. Protocols that did not result in a final systematic review were excluded.

### 4.3 Search methods for identification of studies

Two authors (L. P. G. and Z. P. W.) independently searched the CNKI,^1^ WanFang,^2^ and VIP^3^ databases to identify all the completed systematic reviews published in Chinese journals between January 2009 and December 2022. The deadline for data retrieval was December 2, 2022. The search strategy was as follows:

篇名（词）=系统评价 OR元分析 OR 荟萃分析 OR 元综合,文献类型=论文，年=2009 – 2022

(Title = systematic review OR meta‐analysis OR systematic literature review, Document types = article, Publication date = 2009–2022).

### 4.4 Data collection and analysis

#### 4.4.1 Selection of studies

Before the formal selection, we identified and removed the duplicates using Endnote 20.2.1. Two stages of formal selection existed. In the first stage, two reviewers (J. J. R. and X. Y. H.) independently scanned all titles and abstracts using Rayyan.^4^ In case of discrepancies, we performed two rounds of selection with 50 records before the formal screening until the consistency between them reached 97%. In the second stage, the same two reviewers located the potentially relevant reviews and screened the full text independently based on our criteria. Any disagreements were resolved through consultation with the third reviewer (X. X.).

#### 4.4.2 Data extraction and management

The extraction process comprised two steps. The first part contains publication information, including the first author, title, publication year, and source of the literature. The other part consisted of the study content characteristics, including 9 sections and 38 items (9 sections included the study field, study design, title, abstract, introduction, method, result, discussion, and other information). Details are provided in Supporting Information S1: Appendix A.

Before the formal extraction, four authors (J. J. R., X. Y. H., X. X., and L. P. G.) independently conducted three rounds of pilot extraction with 30 studies based on the data extraction form using Microsoft Excel 2019, until we reached a consensus on all extraction items. In the formal stage, the authors were divided into two groups, and each group extracted data independently. To ensure consistency between the two groups, we randomly selected one eligible review for every 20 articles to be extracted by the four reviewers. Any disagreements within the group were resolved through discussions among all the extractors.

#### 4.4.3 Quality appraisal

Four authors (J. J. R., X. Y. H., X. X., and L. P. G.) evaluated the reporting completeness of the intervention systematic reviews using the PRISMA2020 guideline and PRISMA for Abstract (PRISMA‐A) checklist, non‐intervention systematic reviews using the MOOSE checklist. Additionally, we assessed the methodological quality of intervention systematic reviews using AMSTAR‐2 and non‐interventional systematic reviews using the DART tool. Before the formal evaluation, we independently conducted three rounds of pilot assessments with 30 studies to ensure agreement on all items. During the formal stage, the authors were split into two groups and data were independently extracted. To maintain consistency, we randomly selected an eligible review for every 20 articles for quality assessment using these four extractors. Disagreements within the group were resolved through discussions among all the extractors. The items for each assessment tool are presented in the protocol (Guo et al., 2022).

#### 4.4.4 Data synthesis

We presented the extracted data and summarized the quality assessments as frequencies and percentages for dichotomous data as well as mean and standard deviation (SD) for continuous data. To examine how completeness of reporting affect methodology quality, we determined the correlation coefficient and *p*‐value to test for the relationship between reporting completeness and methodological quality.

#### 4.4.5 Moderator analysis

As per our protocol, we conducted subgroup analyses with stratification analysis and regression analysis to investigate whether the source of literature, research field, number of author, publication year and declaration of funding source could impact the completeness of reporting and methodology quality.

### 4.5 Differences between protocol and review

Compared to our protocol, several adjustments were made during data screening, extraction, and analysis.

For the detailed aims of our present study, we modified our third objective to analyze the potential factors that may influence quality of intervention and non‐intervention systematic reviews published in Chinese social science journals using regression analysis.

At the data screening stage, we eliminated reviews that contained non‐traditional meta‐analyses such as cross‐sectional meta‐analysis, cumulative meta‐analysis, Bayesian meta‐analysis, meta‐regression, meta‐analytic structural equation modelling, and network meta‐analysis. We also removed reviews that primarily focused on identifying and verifying instrument validity.

During the data extraction stage, we divided the reviewers into two groups to manage the numerous reviews included in this study (including the quality assessment process). Due to the high subjectivity and heterogeneity in the overall evidence confidence when using the DART tool, and the controversial contribution of individual items to the overall score in AMSTAR‐2, especially in social science research, we did not assess the overall methodological quality.

At the data analysis stage, we regarded the publication year as dichotomous when assessing the effect of publication year. In addition to the factors mentioned in the protocol, we conducted regression analysis based on the number of authors and funding sources. We adopted regression analysis to explore the effects of categorical factors on overall reporting completeness and methodological quality. If the *p* value is blow 0.05, we proceeded with an ANOVE analysis and post hoc tests (for factors encompassing more than two categories) to determine the direction of effect. All the data analyses were performed using Microsoft Excel 2019 and SPSS26.0.

## 5 RESULTS

### 5.1 Description of studies

#### 5.1.1 Results of the search

**Figure 1:**
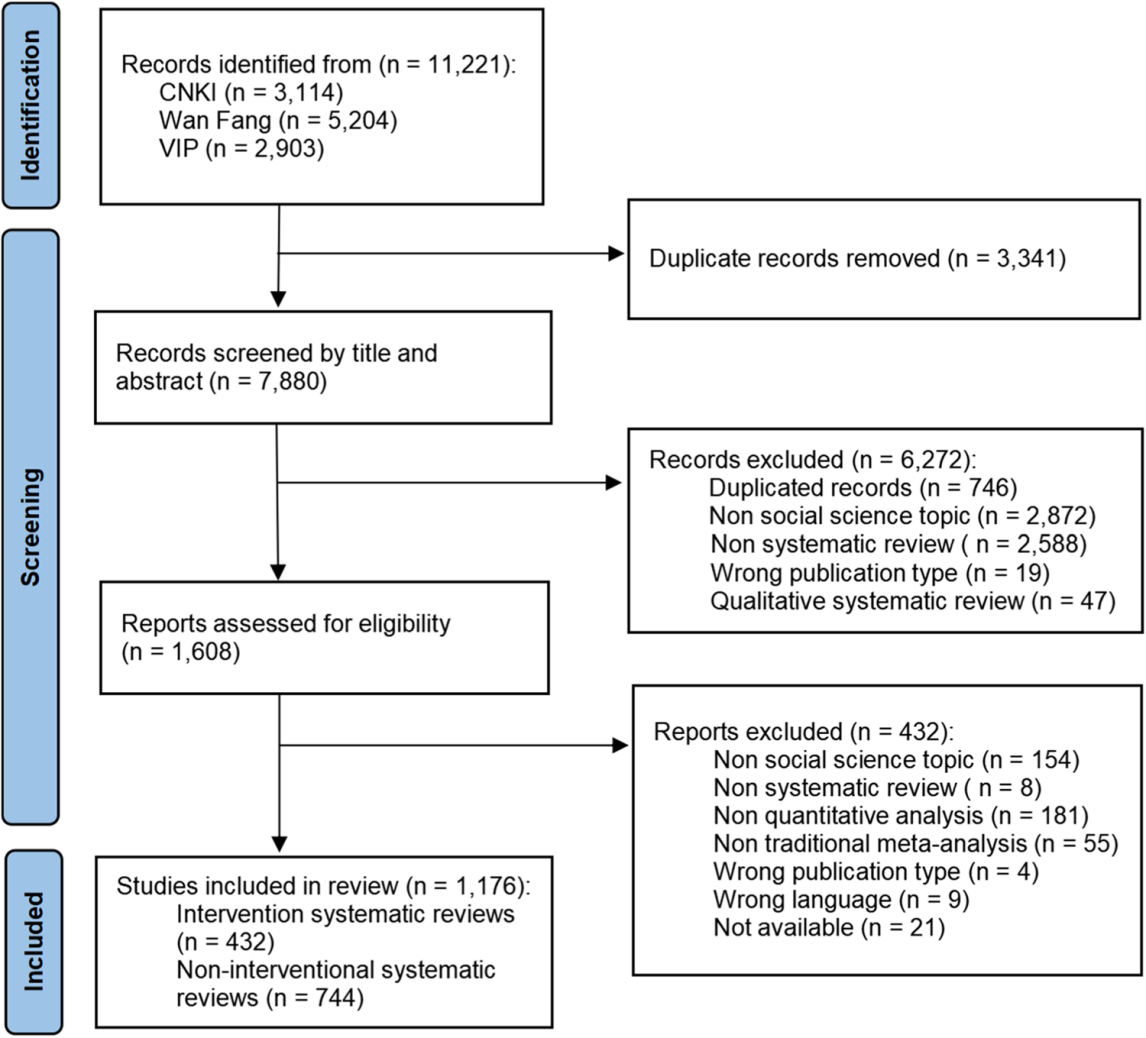
The PRISMA flow for the process of data screening.

The screening process involved double‐screening 7880 titles and abstracts out of the 11,221 studies identified, selecting 1608 full‐text reviews, and 1176 systematic reviews were included. Figure 1 outlines the screening process.

#### 5.1.2 Included studies

Between January 2009 and December 2022, 432 intervention systematic reviews and 744 non‐intervention reviews were published in the social sciences field (Table 1). Of these, 497 (43.1%) were published in journals included in the Chinese Social Science Citation Index (CSSCI). The 1048 reviews were distributed across 10 disciplines, with psychology (375, 35.8%), education (359, 34.3%), and management science (214, 20.4%) as the top three fields of publication. Education had the most intervention reviews (317, 76.9%), while psychology (298, 456.9%) and management science (212, 33.3%) had the most non‐intervention reviews. The majority (751, 71.7%) of reviews reported funding information. The average number of team members involved in these funded reviews was similar to those in reviews did not funding information (3.33 vs. 2.90 people). However, 92 reviews (8.8%) were conducted by single author and 48 of these reviews received funding.

**Table 1:** Epidemiological characteristics of the included studies (1176 reviews).

Characteristics	Category	Number (%)
Type of systematic review	Intervention	432 (36.7)
	Non‐intervention	744 (63.3)
Year of publication	2009	16 (1.4)
	2010	20 (1.7)
	2011	33 (2.8)
	2012	38 (3.2)
	2013	34 (2.9)
	2014	54 (4.6)
	2015	65 (5.5)
	2016	80 (6.8)
	2017	97 (8.2)
	2018	104 (8.8)
	2019	149 (12.7)
	2020	144 (12.2)
	2021	168 (14.3)
	2022	174 (14.8)
Type of journal	CSSCI	497 (42.3)
	Non‐CSSCI	679 (57.7)
Discipline	Psychology	417 (35.5)
	Education	388 (33.0)
	Management Science	263 (22.4)
	Sociology	30 (2.6)
	Library Information Science	27 (2.3)
	Linguistics	15 (1.3)
	Economics	12 (1.0)
	Journalism and Communication Studies	11 (0.9)
	Political science	11 (0.9)
	Law	2 (0.2)
Number of authors	1	115 (9.8)
	2	362 (30.8)
	3	318 (27)
	4	186 (15.8)
	5	97 (8.2)
	6	54 (4.6)
	≥7	44 (3.7)
Number of included studies	1–10	157 (13.4)
	11–20	275 (23.4)
	21–30	197 (16.8)
	31–40	167 (14.2)
	41–50	115 (9.8)
	51–60	86 (7.3)
	≥61	176 (15.0)
	not reported	3 (0.26)
Number of databases searched	0	4 (0.3)
	1	77 (6.5)
	2	102 (8.7)
	3	212 (18)
	4	148 (12.6)
	5	142 (12.1)
	6	121 (10.3)
	7	119 (10.1)
	≥8	233 (19.8)
	not reported	18 (1.5)
PRISMA citation	yes	29 (2.5)
	no	1147(97.5)
Eligible study designs		
Intervention reviews (432)	RCT	229 (19.5)
	Non‐RCT	36 (3.1)
	Quasi‐experiment	88 (7.5)
	Cohort study	4 (0.3)
	Case‐controlled study	4 (0.3)
	Other control trail	74 (6.3)
	Other randomized trial	6 (0.5)
	No specified experimental study	75 (6.4)
	Not reported	46 (3.9)
Non‐intervention reviews (744)	Cohort study	13 (1.1)
	Case‐controlled study	10 (0.9)
	Cross‐sectional study	70 (6.0)
	No specified empirical study	11 (0.9)
	Not reported	405 (34.4)

Abbreviations: B, unstandardized coefficients; Beta, standardized coefficients; CI, confidence interval; LL, lower limits; *P*, *p*‐value; SE, standard error of unstandardized coefficients; t, t‐test; UL, uper limits; VIF, variance inflation factor.

The number of primary studies included in these reviews ranged from 3 to 301 (median = 27), and the sample sizes ranged from 11 to 2,835,969 (median = 10,196).

Out of the total reviews, only 27 (2.6%) explicitly mentioned using the PRISMA guideline. Among these, 14 reviews solely provided the PRISMA flow diagram, one review cited PRISMA for protocol (PRISMA‐P), and 12 reviews complied with PRISMA reporting statement. Detailed information is provided in Supporting Information S2: Appendix B.

Citations and additional coding of the included reviews are available in supporting information Supporting Information S2: Appendix B.

### 5.2 Completeness of reporting assessment

Intervention and non‐intervention systematic reviews were evaluated for reporting completeness using PRISMA2020 and MOOSE guidelines, respectively.

#### 5.2.1 Reporting completeness of intervention systematic reviews

##### Reporting completeness of title and abstract

***Title.*** Out of 432 reviews, only 50 (11.6%) were named ‘systematic review’ in the title, including six being described as ‘systematic review and meta‐analysis’, and the majority of reviews were labelled as ‘meta‐analysis’ (382, 88.4%).

**Figure 2:**
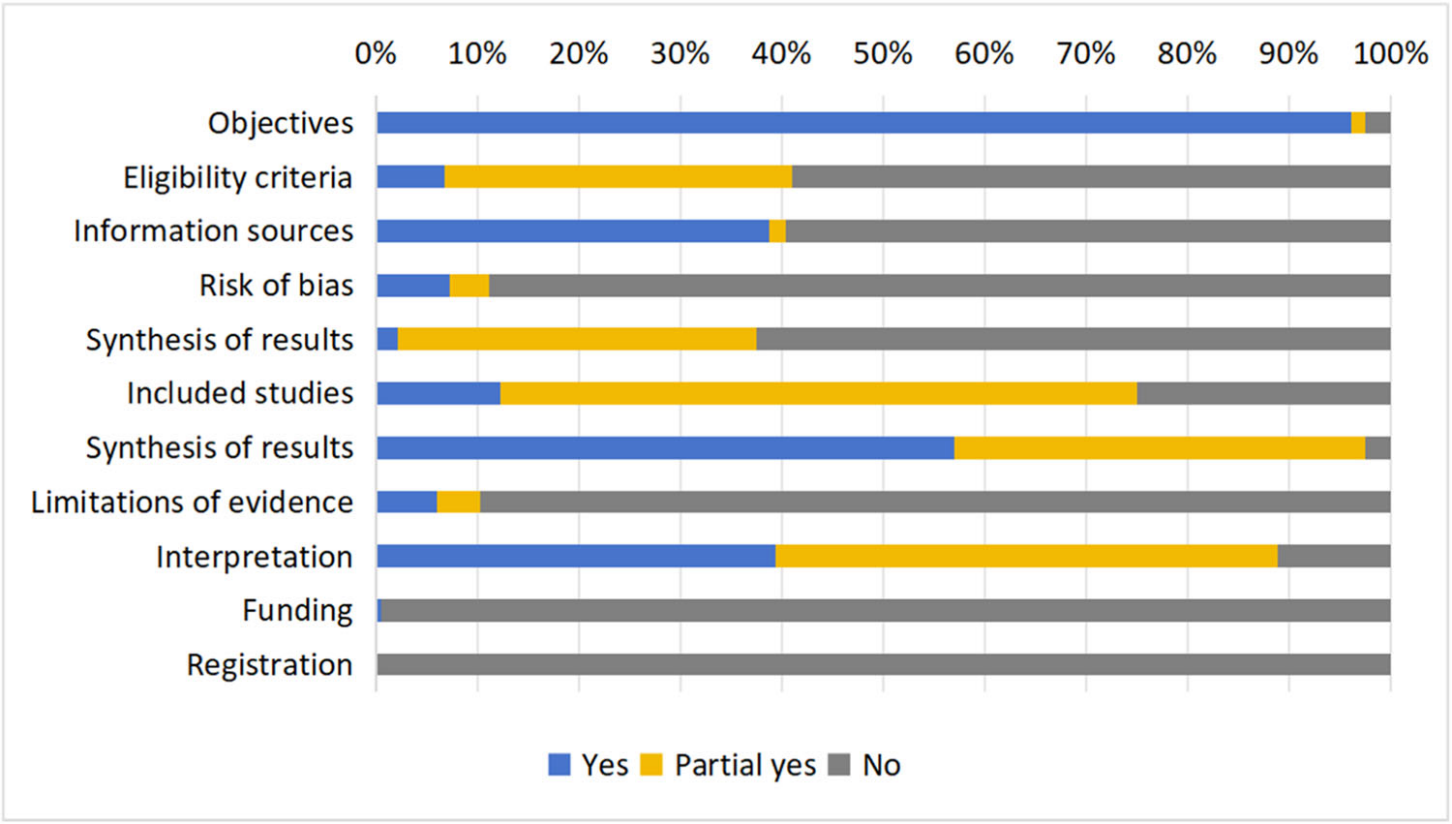
Percentage of PRISMA‐A reported in intervention reviews (432 reviews).

***Abstract.*** None review reported all 11 items in the PRISMA‐A checklist, and the median number of items reported was five. The overall reporting completeness of the abstracts was 45.4%, with 21.2% of items partially reported. The reviews had a high level of reporting completeness on their background (objectives), and the studies included the synthesis and interpretation of results. There are 67.6% of the reviews reported the methods used (including eligibility criteria, information sources, and synthesis of results), whereas only 11.1% reported the methods used to assess the risk of bias. None of the reviews mentioned the registration information in the abstract (see Figure 2).

##### Reporting completeness of full‐text

**Figure 3:**
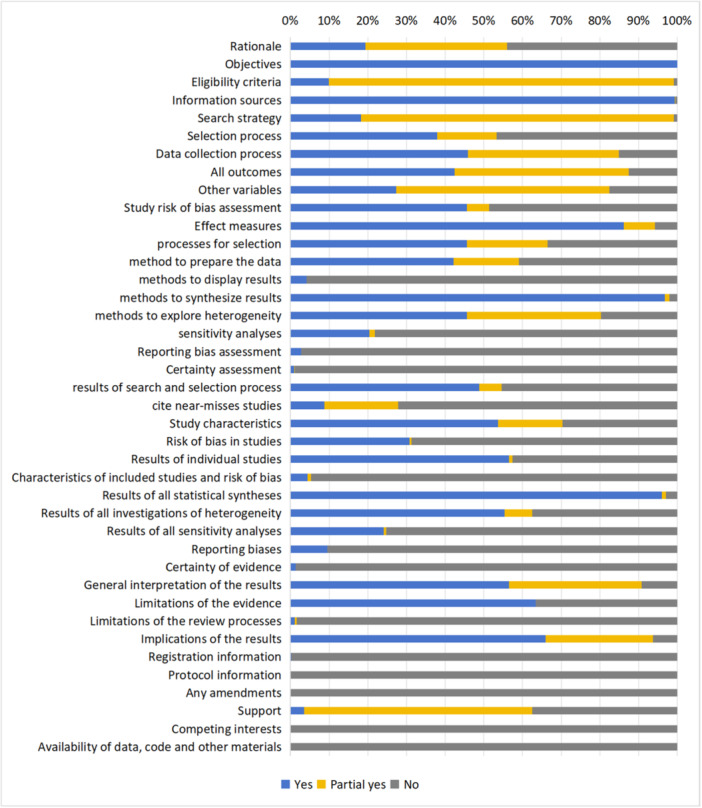
The percentage of PRISMA reported in intervention reviews (432 reviews). The details of each item were shown in protocol (Guo et al., 2022).

***Introduction.*** The intervention reviews had an average reporting completeness rate of 49.9%. Figure 3 shows that all reviews stated the objective(s) or question(s) they addressed, whereas 56.0% described their rationale based on existing knowledge.

***Method and results.*** Most of included reviews reported the eligibility criteria (428, 99.1%), and 98.8% were based on the Population, Intervention, Comparison, Outcome, and Setting (PICOS) framework. Majority of reviews reported their literature search process (428, 99.1%); however, only 18.3% provided a full search strategy. Half of the reviews described the method and results of the selection process (216, 50%) and 43.5% specified the number and reasons for excluding studies during full‐text screening. More than 80% of the studies listed their data collection process and all the items extracted, and 68.5% cited each included study and presented its characteristics.

Although 222 reviews (51.4%) outlined the process of evaluating risk of bias in the primary studies, 135 reported the results accordingly. Additionally, only 41 (9.5%) of the reviews identified reporting biases, and 6 (1.4%) assessed the certainty of evidence and shared their findings.

Of all the meta‐analyses conducted, 97.9% described the methods used to synthesize the data and reported statistical results. Meanwhile, 80.3% of the reviews mentioned the methods used to explore the potential causes of heterogeneity among the study results, but 77 (17.8%) did not report their results accordingly. Only 107 studies (24.8%) conducted a sensitivity analysis to evaluate the robustness of the synthesized results.

***Discussion and other information.*** Most reviews (over 90%) provided a general interpretation of the results in the context of other evidence and discussed the implications of the results for practice, policy, and/or future research. Additionally, 63.4% of reviewers discussed the limitations of the evidence included in their review. However, only 7 (1.6%) discussed the limitations of the review process.

Of the 432 systematic reviews, 62.5% cited sources of financial support. However, none of these reviews provide information on the specific role of the funder or sponsors in the review process. Few reviews mentioned that the registration information disclosed conflicts of interest among the authors or indicated the availability of data, codes, or other materials.

#### 5.2.2 Reporting completeness of non‐intervention systematic reviews

**Figure 4:**
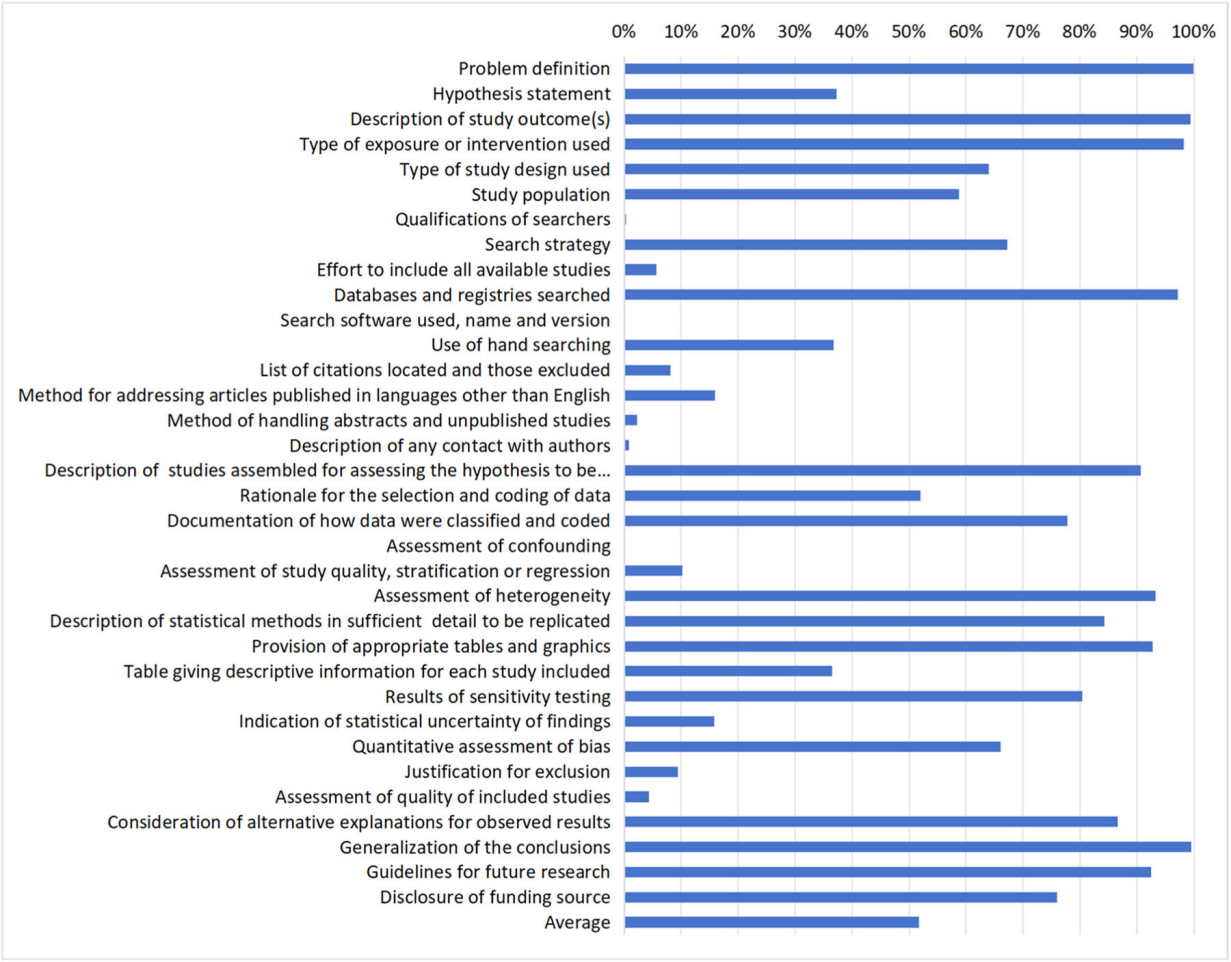
The percentage of MOOSE items reported in non‐intervention reviews (744 reviews). The details of each item were shown in protocol (Guo et al., 2022).

Out of 744 non‐intervention reviews, the majority reviews (739, 99.3%) were identified as ‘meta‐analysis’, and only 4 were named ‘systematic review’; 1 was reported as a ‘systematic review and meta‐analysis’. The non‐intervention reviews had an average reporting completeness of 51.8%, and the reporting completeness of each item in the MOOSE is shown in Figure 4.

***Background.*** All reviews defined the problems they addressed and reported the eligibility criteria, including population (437, 58.7%), type of exposure (731, 98.3%, outcomes (740, 99.5%), and study design (476, 64.0%). Over one‐third of the reviews (277, 37.2%) proposed a hypothesis before performing the data analysis.

***Search Strategy.*** Most of included reviews reported the search strategy, including the retrieved database resources (722, 97.0%) and search keywords (679, 91.2%). Over one‐third (274, 36.8%) of the non‐intervention reviews reported hand searching; among them, 192 searched the reference lists, 78 screened the related journals, 183 searched the web page, 29 contacted experts related to the topic, and 30 conducted other grey literature search. Only two reviews described the involvement of librarians in the literature search, and none reported the search software used, name, or version. Few reviews (42, 5.6%) reported the process of contacting authors, and only six described the results obtained from all the studies by the authors.

Majority of reviews reported the inclusion criteria (705, 94.8%), but few addressed the method for articles published in languages other than English/Chinese (119, 16.0%) or the method of handling abstracts and unpublished studies (17, 2.3%).

***Methods and results.*** Nearly 80% of the reviews listed the extracted items (579, 77.8%), 387 (52.0%) described the process of screening and data coding, 73 (11.5%) performed the selection process dependently, and 306 (41.1%) conducted data extraction by at least two reviewers. However, only 272 (36.6%) reviews presented descriptive information for each included study.

As for bias assessment, only one study considered confounding factors, 76 (10.2%) evaluated the study quality, and 492 (66.1%) conducted a publication bias assessment using a quantitative method. Most reviews (694, 93.3%) assessed heterogeneity and explored the potential moderators with subgroup analysis (566, 83.2%) and robustness tests of the overall effect (112, 16.4%).

Most of reviews (623, 83.7%) reported the method for data synthesis and their justification; all reviews reported the results of the meta‐analysis, and 271 (36.4%) presented the individual results with tables or graphics.

***Discussion and conclusion.*** Based on the results of the meta‐analysis,636 (85.5%) reviews provided an interpretation of the results, with 405 (54.4%) reviews illustrating the observed results in the context of other evidence. Few studies explained the exclusion criteria for language restrictions (70, 9.4%) and discussed the impact of study quality on the overall results (32, 4.3%).

Most reviews (741, 99.6%) drafted a general conclusion, and over 90% discussed the implications of the results for future research. Nearly 80% of the reviews disclosed the funding source, but none described the role of the funder.

### 5.3 Methodology quality assessment

The AMSTAR‐2 and DART checklists were used to evaluate the methodological quality of intervention and non‐intervention reviews, respectively.

#### 5.3.1 Methodology quality of intervention systematic reviews

**Figure 5:**
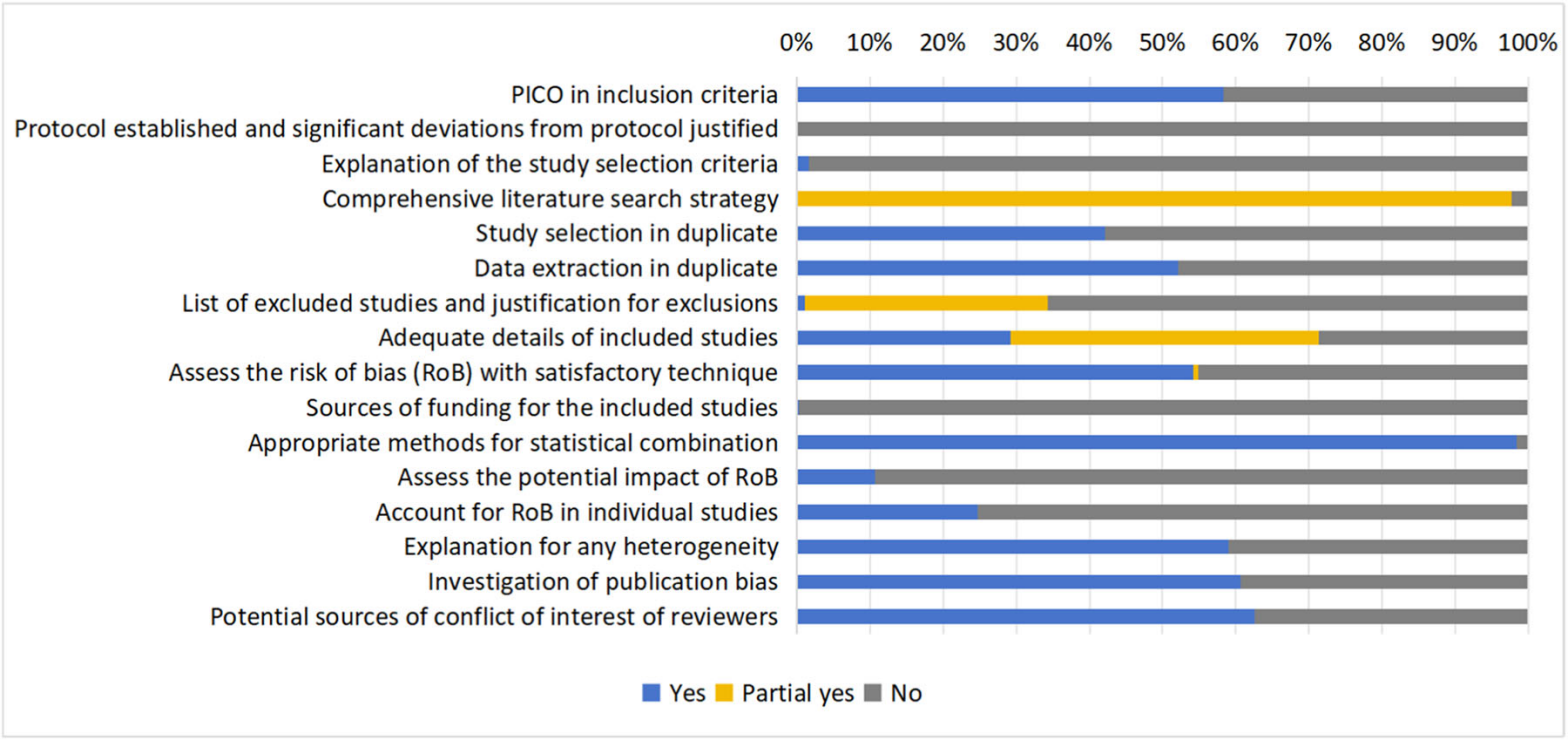
AMSTAR‐2 assessment of each item (432 intervention reviews).

Nine of the sixteen AMSTAR‐2 items were completely or partially addressed in more than half of the reviews (Figure 5). Almost of all reviews addressed the literature search (430, 99.5%), but none retrieved all the sources recommended in the AMSTAR‐2 checklist, including 3 studies did not mentioned the databases searched, and 17 reviews only searched a single database. Few reviews emphasized registration (0.0%), rationality of the study selection criteria (1.6%), sources of funding for primary studies (0.2%), and methods to assess the impact of risk of bias of individual studies on the results of the meta‐analysis (10.6%).

#### 5.3.2 Methodology quality of non‐intervention systematic reviews

**Figure 6:**
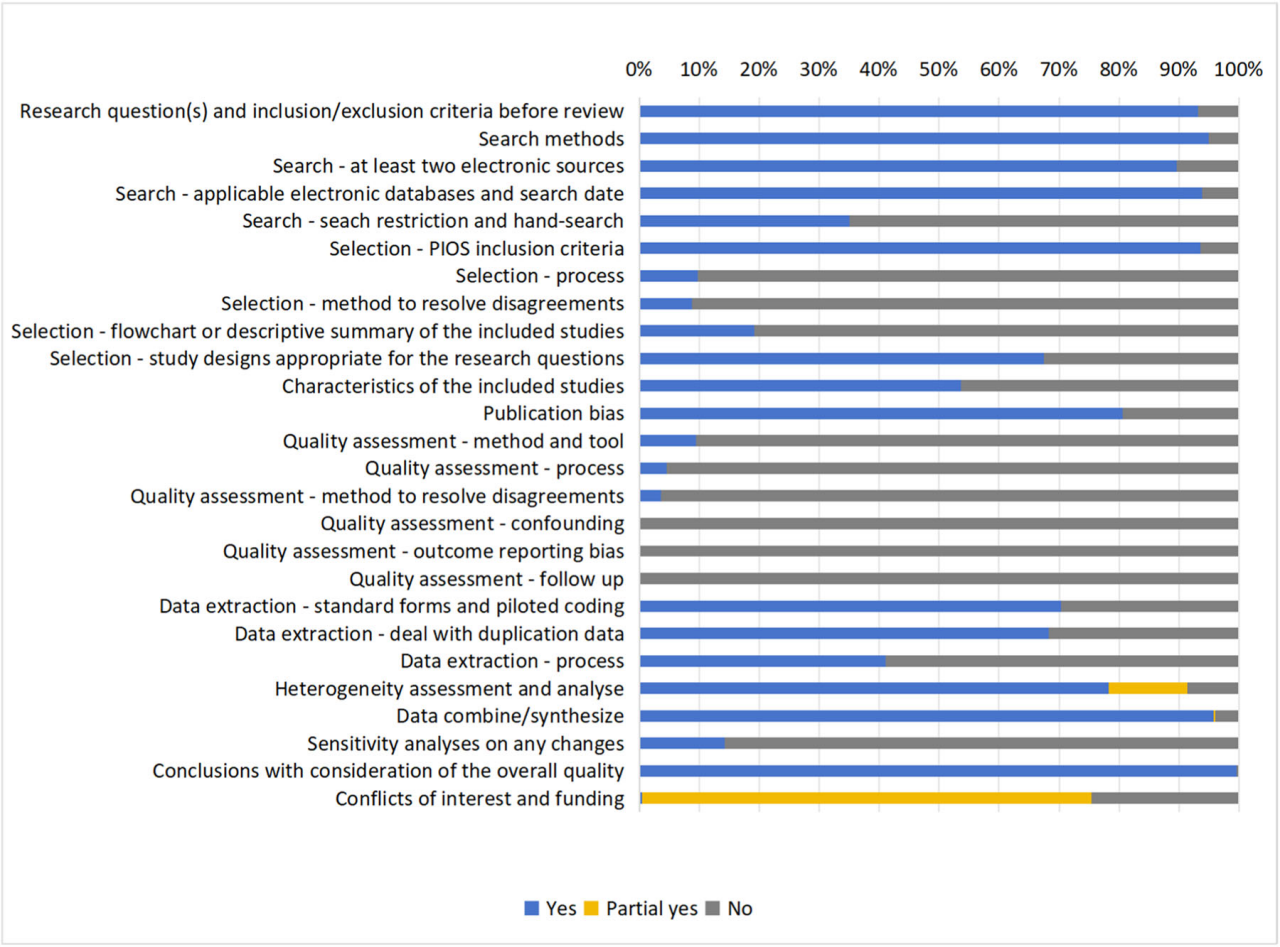
DART assessment of each item (744 non‐intervention reviews).

Although the majority of reviews addressed key procedures for conducting systematic reviews, many lacked the details on these following processes such as research development of the research question, literature search and selection, assessment of publication bias, data extraction and synthesis, and heterogeneity analysis; for example, 64.9% of reviews did not conduct a manual search, nearly 90% of reviews did not report the process and results of selection, and over half of the reviews (58.9%) did not describe the process of data extraction. Only 143 (19.2%) reviews presented the results of the search and screening processes with a flow diagram, and 132 (17.7%) listed the number of excluded studies and their justifications (Figure 6).

Few reviews (71, 9.5%) assessed the quality of the included studies, and only 52 reviews reported the results of overall quality, while none of the reviews examined bias by confounding factors, outcome reporting bias, and loss to follow‐up. Nearly 80% of the reviews reported funding, but only three of them clarified the conflict of interests in which the author(s) were involved.

### 5.4 The consistency between reporting completeness and methodological quality

Table 2 shows that higher reporting completeness of intervention reviews is associated with better methodological quality, especially for full reviews (PRISMA‐A: *β* = 0.25, *p* < 0.00; PRISMA: *β* = 0.67, *p* < 0.00). Table 3 also indicates that reporting completeness positively affects methodological quality of non‐intervention reviews (*β* = 0.68, *p* < 0.00). More details are provided in Supporting Information S3: Appendix C.

**Table 2:** The regression analysis of reporting completeness and methodological quality of intervention reviews (432 reviews).

						95% CI		
Factors	B	SE	Beta	*t*	*P*	LL	UL	VIF
Average reporting completeness of abstract (PRISMA‐A)								
Constant	−4.87	4.69	.	−1.04	0.30	−14.09	4.36	.
Source of literature	−0.11	0.02	−0.33	−6.92	<0.00	−0.14	−0.08	1.11
Number of authors	0.02	0.00	0.17	3.72	<0.00	0.01	0.03	1.08
Publication year	0.00	0.00	0.05	1.13	0.26	0.00	0.01	1.06
Research field	0.00	0.01	0.00	0.04	0.97	−0.01	0.01	1.03
Funding source declared	−0.01	0.02	−0.03	−0.54	0.59	−0.04	0.02	1.15
*Adjusted R^2^*				0.14				
*Significance*				<0.00				
*Standard error of the estimate*				0.14				
Average reporting completeness of full review (PRISMA)								
Constant	−15.32	2.88	.	−5.32	<0.00	−20.99	−9.66	
Source of literature	−0.05	0.01	−0.25	−5.37	<0.00	−0.07	−0.03	1.11
Number of authors	0.01	0.00	0.21	4.63	<0.00	0.01	0.02	1.08
Publication year	0.01	0.00	0.24	5.46	<0.00	0.01	0.01	1.06
Research field	0.01	0.00	0.06	1.40	0.16	0.00	0.01	1.03
Funding source declared	0.03	0.01	0.14	2.93	<0.00	0.01	0.05	1.15
*Adjusted R^2^*				0.19				
*Significance*				<0.00				
*Standard error of the estimate*				0.20				
Average methodological quality (AMSTAR‐2)								
Constant	−16.56	4.29	.	−3.86	<0.00	−24.99	−8.12	
Source of literature	−0.10	0.02	−0.30	−6.59	<0.00	−0.13	−0.07	1.11
Number of authors	0.02	0.00	0.18	4.05	<0.00	0.01	0.02	1.08
Publication year	0.01	0.00	0.17	3.95	<0.00	0.00	0.01	1.06
Research field	−0.01	0.01	−0.04	−0.90	0.37	−0.02	0.01	1.03
Funding source declared	0.07	0.01	0.22	4.89	0.00	0.04	0.10	1.15
*Adjusted R^2^*				0.21				
*Significance*				<0.00				
*Standard error of the estimate*				0.13				
Methodological quality (AMSTAR‐2) and reporting completeness (PRISMA‐A and PRISMA)								
Constant	−0.14	0.02		−6.20	<0.00	−0.19	−0.10	
PRISMA‐A	0.17	0.03	0.18	5.35	<0.00	0.11	0.23	1.19
PRISMA	1.05	0.05	0.70	21.42	<0.00	0.95	1.15	1.19
*Adjusted R^2^*				0.61				
*Significance*				<0.00				
*Standard error of the estimate*				0.91				

Abbreviations: B, unstandardized coefficients; Beta, standardized coefficients; CI, confidence interval; LL, lower limits; *P*, *p*‐value; SE, standard error of unstandardized coefficients; t, t‐test; UL, uper limits; VIF, variance inflation factor.

**Table 3:** The regression analysis of reporting completeness and methodological quality of non‐intervention reviews (744 reviews).

						95% CI		
Factors	B	SE	Beta	*t*	*P*	LL	UL	VIF
Average reporting completeness (MOOSE)								
Constant	−17.47	1.67	.	−10.45	<0.00	−20.76	−14.19	.
Source	0.02	0.01	0.11	3.27	<0.00	0.01	0.03	1.12
Authors	0.01	0.00	0.20	6.12	<0.00	0.01	0.02	1.06
Year	0.01	0.00	0.34	10.69	<0.00	0.01	0.01	1.08
Field	0.00	0.00	−0.01	−0.28	0.78	0.00	0.00	1.12
Funding source declared	0.05	0.01	0.23	6.69	<0.00	0.03	0.06	1.18
*Adjusted R^2^*				0.29				
*Significance*				<0.00				
*Standard error of the estimate*				0.08				
Average methodological quality (DART)								
Constant	−22.16	2.40		−9.24	<0.00	−26.87	−17.45	
Source	0.00	0.01	0.00	0.00	1.00	−0.02	0.02	1.12
Authors	0.03	0.00	0.30	9.37	<0.00	0.02	0.04	1.06
Year	0.01	0.00	0.30	9.39	<0.00	0.01	0.01	1.08
Field	0.00	0.00	−0.06	−1.68	0.09	−0.01	0.00	1.12
Funding source declared	0.06	0.01	0.20	5.74	<0.00	0.04	0.08	1.18
*Adjusted R^2^*				0.28				
*Significance*				<0.00				
*Standard error of the estimate*				0.11				
Methodological quality (DART) and reporting completeness (MOOSE)								
Constant	0.25	0.01	.	21.29	<0.00	0.23	0.27	.
MOOSE	0.50	0.02	0.68	23.25	<0.00	0.46	0.54	1.00
*Adjusted R^2^*				0.46				
*Significance*				<0.00				
*Standard error of the estimate*				0.06				

Abbreviations: B, unstandardized coefficients; Beta, standardized coefficients; CI, confidence interval; *P*, *p*‐value; SE, standard error of unstandardized coefficients; VIF, variance inflation factor.

### 5.5 Factors that influence the reporting completeness and methodological quality

#### 5.5.1 Factors that influence intervention systematic reviews

***Source of literature.*** The source of the literature was a significant predictor of reporting completeness and methodological quality of intervention reviews as measured by the PRISMA and AMSTAR tools. Table 2 shows that intervention reviews published in journals indexed in CSSCI had lower compliance rates on PRISMA‐A, PRISMA, and AMSTAR‐2 than those published in other journals (PRISMA‐A: 37.0% vs. 48.8%, *β* = −0.33, *p* < 0.00; PRISMA: 46.7% vs. 51.1%, *β* = −0.25, *p* < 0.00; AMSTAR‐2: 39.6% vs. 47.9%, *β* = −0.30, *p* < 0.00).

***Number of authors.*** As shown in Table 2, the number of authors was positively associated with reporting completeness and methodological quality of the intervention reviews. Specifically, intervention reviews conducted by four or more authors had higher compliance rates for PRISMA and AMSTAR‐2 than those conducted by single or two authors (see Supporting Information S3: Appendix C for details).

**Figure 7:**
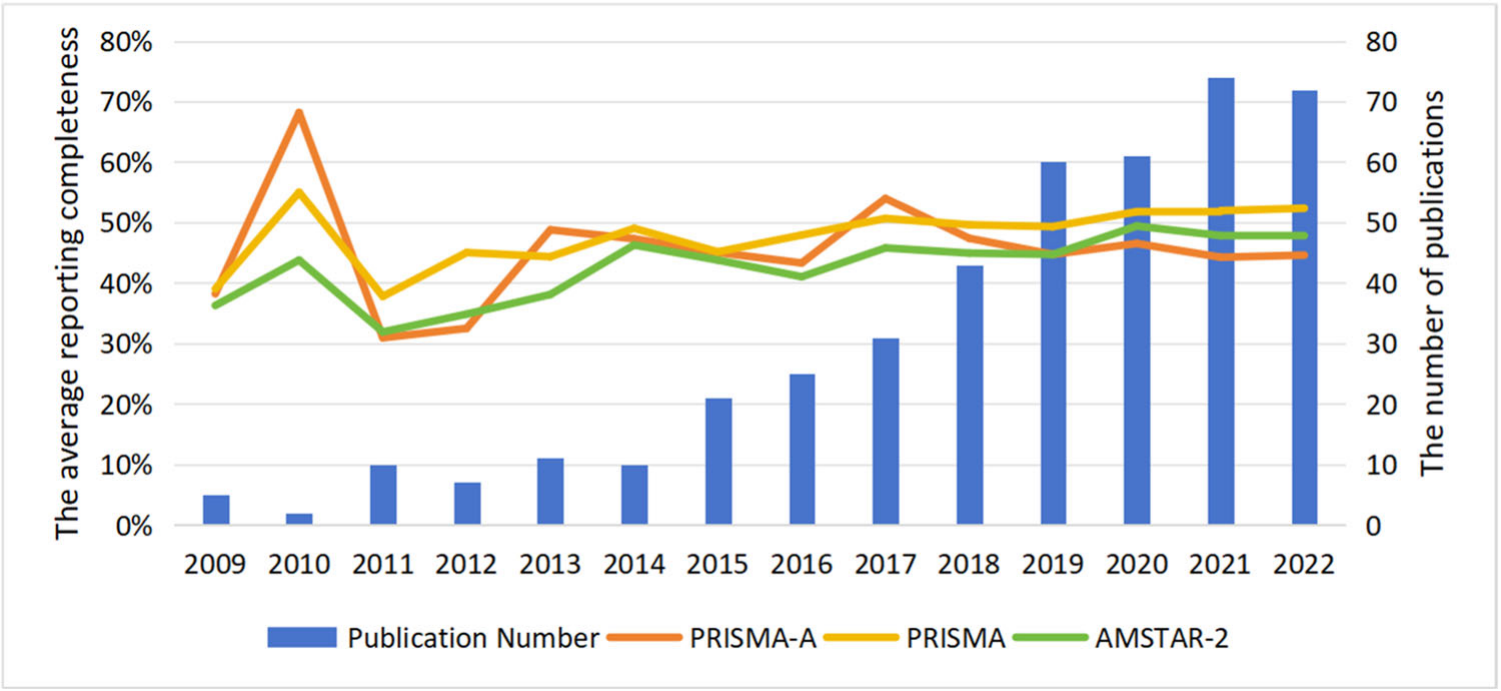
The average scores of intervention review by year.

**Figure 8:**
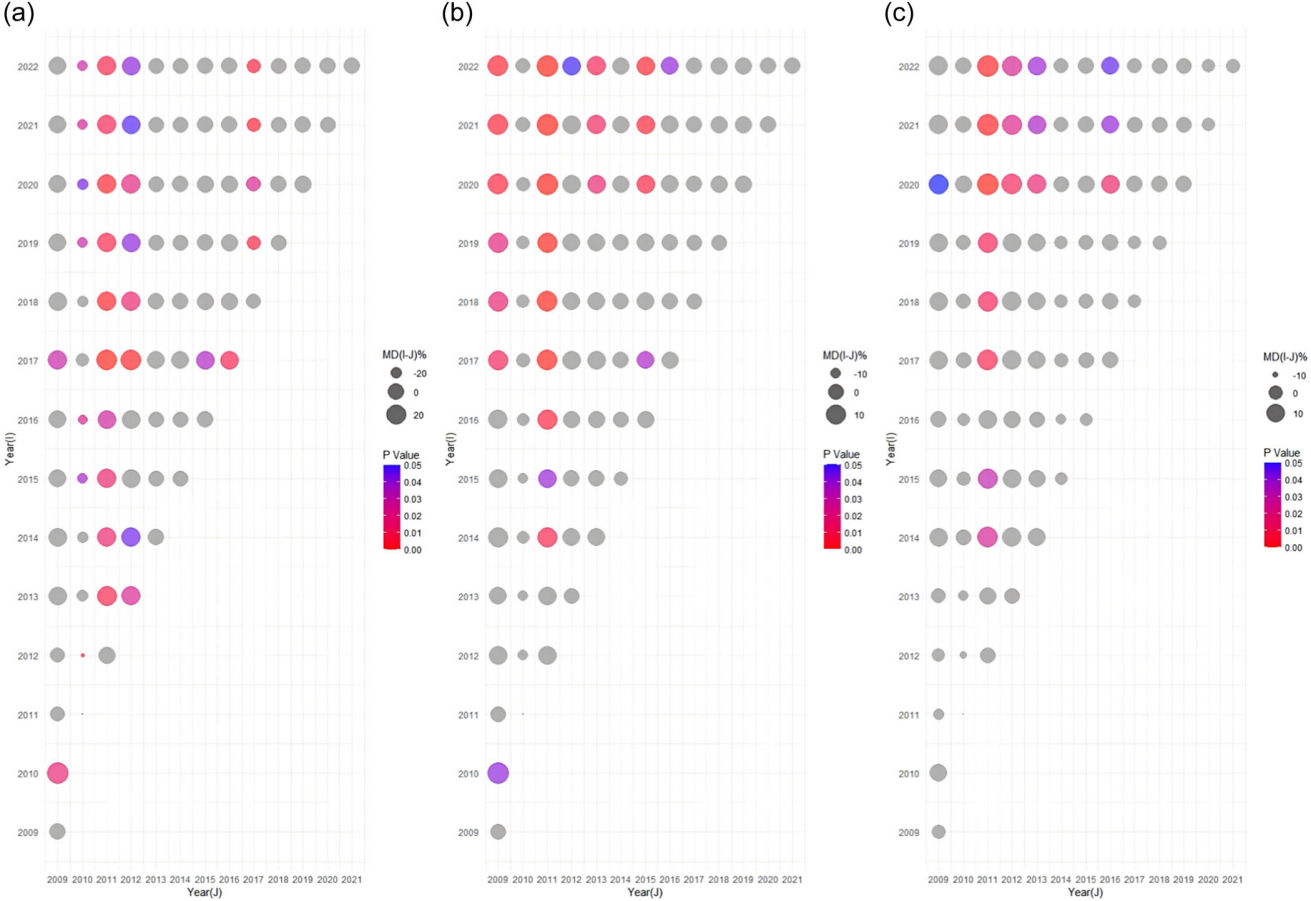
Multiple comparisons of reporting completeness and methodological quality in intervention reviews. (a) multiple comparisons of PRISMA‐A; (b) multiple comparisons of PRISMA; (c) multiple comparisons of AMSTAR; MD, Mean difference of reporting completeness.

***Publication year.*** Publication year was also a significant factor affecting the reporting completeness and methodological quality of the intervention reviews. Figure 7 illustrates the increasing trend in systematic intervention reviews and meta‐analyses in social science research since 2014, with more than 70 papers published in 2021. Table 2 reveals that the compliance rates on PRISMA and AMSTAR‐2 increased with the publication year (PRISMA: *β* = 0.24, *p* < 0.00; AMSTAT‐2: *β* = 0.17, *p* < 0.00), particularly noticeable since 2020. However, the reporting completeness on PRISMA‐A remained stable (PRISMA‐A: *β* = 0.05, *p* = 0.26). The details of the multiple comparisons by year were shown in Figure 8.

***Research field.*** This was not a significant factor influencing the reporting completeness or methodological quality of the intervention reviews. The 432 intervention reviews were distributed across 9 research fields, including education (*n* = 331, 76.6%), psychology (*n* = 83, 19.2%), linguistics (*n* = 8, 1.9%), sociology (*n* = 3, 0.7%), management science (*n* = 2, 0.5%), library information science (*n* = 1, 0.2%), journalism and communication studies (*n* = 1, 0.2%), political science (*n* = 2, 0.5%), and law (*n* = 1, 0.2%). Table 2 shows that the compliance rates for PRISMA‐A, PRISMA, and AMSTAR‐2 were similar across the fields.

***Declaration of funding sources.*** This was another significant predictor of reporting completeness and methodological quality of intervention reviews. Table 2 demonstrates that the 270 intervention reviews that declared their funding sources had higher compliance rates on PRISMA and AMSTAR‐2 than those that did not disclose their funding sources (PRISMA: 51.2% vs. 47.7%, *β* = 0.14, *p* < 0.00; AMSTAR‐2: 48.1% vs. 41.2%, *β* = 0.22, *p* < 0.00). However, there was no significant difference in the compliance rates on PRISMA‐A between the two groups (PRISMA‐A: 44.7% vs. 46.5%, *β* = −0.03, *p* = 0.59).

#### 5.5.2 Factors that influence on non‐intervention systematic reviews

***Source of literature.*** In contrast to intervention reviews, non‐intervention reviews from journals indexed in CSSCI had higher reporting completeness than those from other journals (53.3% vs. 50.3%, *β* = 0.11, *p* < 0.00). However, there was no significant difference in the methodological quality (51.0% vs. 50.0%, *β* = 0.00, *p* = 1.00).

***Number of authors.*** Table 3 shows that the number of authors had a significant positive impact on the reporting completeness and methodological quality of non‐intervention reviews (MOOSE: *β* = 0.20, *p* < 0.00; DART: *β* = 0.30, *p* < 0.00). Specifically, single‐author non‐intervention reviews were of lower quality than those produced by two or more authors, and reviews conducted by six or more authors had a higher completeness of reporting (see Supporting Information S2: Appendix B for details).

**Figure 9:**
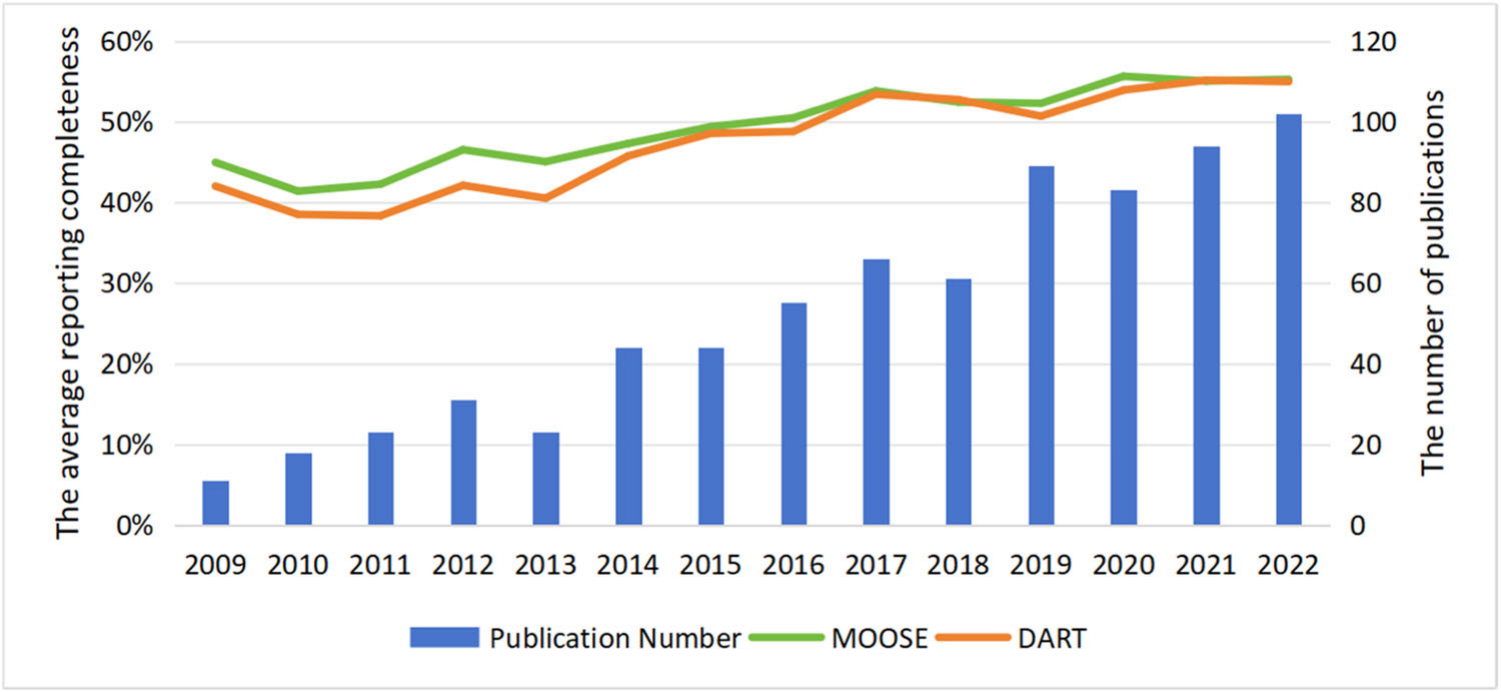
The average reporting completeness of the non‐intervention reviews by year.

**Figure 10:**
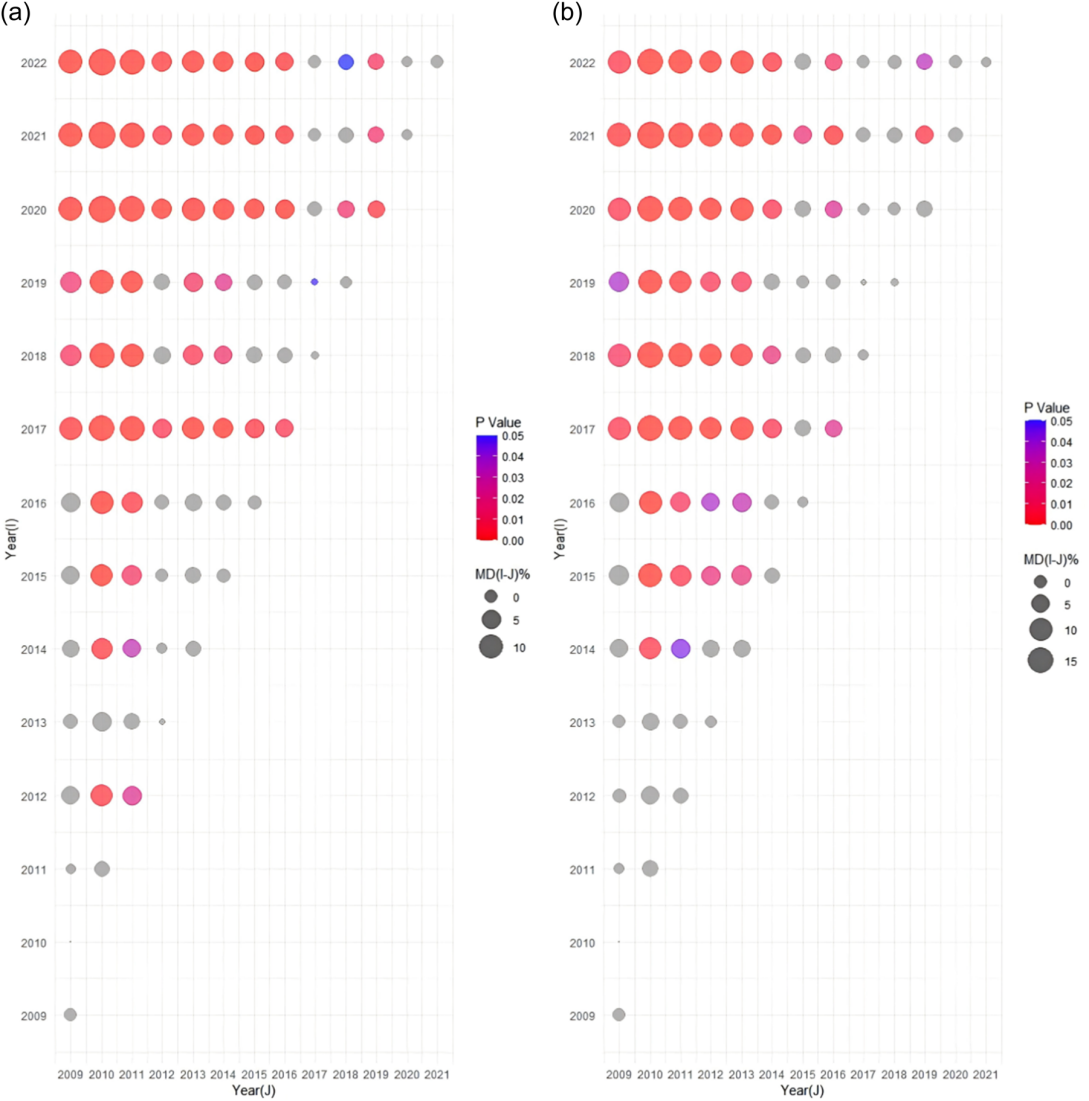
Multiple comparisons of reporting completeness and methodological quality in non‐interventional reviews. (a) multiple comparisons of MOOSE; (b) multiple comparisons of DART; MD, Mean difference of reporting completeness.

***Publication year.*** As shown in Figure 9, the number of non‐intervention reviews in social science research increased since 2014, reaching 96 by 2022. The results of regression analysis showed that the reporting completeness and methodological quality of non‐intervention reviews improved since 2014 (MOOSE: *β* = 0.34, *p* < 0.00; DART: *β* = 0.30, *p* < 0.00). The details were shown in Figure 10.

***Research field.*** The 744 non‐intervention reviews were distributed across 10 research fields, with psychology being the most popular field (*n* = 334, 44.9%), followed by management science (*n* = 261, 35.1%), education (*n* = 57, 7.7%), sociology (*n* = 27, 3.6%), library information science (*n* = 26, 3.5%), economics (*n* = 15, 2.0%), journalism and communication studies (10, 1.3%), political science (*n* = 9, 1.2%), linguistics (*n* = 4, 0.5%), and law (*n* = 1, 0.1%). The reporting completeness and the methodological quality were similar across these fields (MOOSE: *β* = −0.01, *p* = 0.78; DART: *β* = −0.06, *p* = 0.09). Reviews in psychology, education, economics and management had a lower methodological quality than those in library information science, journalism, and communication studies, and Education (see Supporting Information S2: Appendix B for details).

***Declaration of funding sources.*** Out of 744 non‐intervention reviews, 565 (75.9%) disclosed their funding sources, and these reviews had higher reporting completeness and methodological quality than those without financial support (MOOSE: 53.6% vs. 46.0%, *β* = 0.23, *p* < 0.00; DART: 52.7% vs. 43.7%, *β* = 0.20, *p* < 0.00).

## 6 DISCUSSION

### 6.1 Summary of main results

We assessed the reporting completeness and methodological quality of 1176 systematic reviews and meta‐analyses published in Chinese social science journals (497 reviews were indexed in CSSCI), comprising 432 intervention and 774 non‐intervention reviews. More than 90% of these reviews belonged to psychology, education, and management science fields. Half of the reviews had 2 or 3 authors, whereas 115 reviews were conducted by single authors. Only 29 reviews explicitly mentioned using the PRISMA guideline.

#### 6.1.1 Summary of main results on intervention reviews

For intervention reviews, the abstracts and the full reviews had low reporting completeness. They reported well on the title, objectives, the synthesis of results, and the interpretation of results. however, they inadequately reported the risk of bias assessment, full search strategy, reporting biases, certainty of evidence, and sensitivity analysis. For specific characteristics, 49.6% of reviews did not report the method and process of risk of bias assessment, and 41% of the reviews that reported the method did not present the results accordingly. Only 23 reviews considered the potential impact of risk of bias on the overall synthesis results, and 41 reviews discussed the reporting bias due to missing data.

A clear search strategy is one of the criteria for conducting a systematic review. However, most of the included reviews only reported the keywords they used for literature searching, that obstructed future researchers from replicating the results of their reviews based on the reports. Few reviews evaluated the certainty of evidence. The Grading of Recommendations Assessment, Development and Evaluation (GRADE) standards are often used to evaluate the strength of evidence an certainty of conclusions in systematic reviews and meta‐analyses in health care research: however, social sciences research involves diverse and complex phenomena, ethical and practical issues, a lack of standardized methods and tools, and potential bias and influence of researchers, that makes it difficult to apply the standards (Meader et al., 2014).

Only two reviews reported the registration and protocol information. This may be because there is a lack of Chinese registration platforms for systematic reviews in social science, and language is also a barrier for Chinese researchers to register their reviews on other English‐oriented platforms, such as Campbell Collaboration,^5^ Best Evidence in Medical Education,^6^ or Open Science Framework.^7^ The availability of data, code, or other materials is essential for ensuring the transparency, reproducibility, and quality of systematic reviews (South & Lorenc, 2020). However, no review provided availability of data, code, or other materials. This may be due to the lack of incentives, standards, platforms, or policies for data and code sharing, or the concerns about privacy, ethics, or ownership of data and code (Page et al., 2022).

It is not surprising that the methodological quality of intervention systematic reviews was poor, especially on registration, study selection criteria, funding sources, and impact assessment. The methodological quality assessment was performed based on the reported information from the reviews, and most of the items in AMSTAR‐2 were also consistent with the method section of reporting guideline. Therefore, the methodological assessment presented similar results to reporting completeness of the intervention reviews.

#### 6.1.2 Summary of main results on non‐intervention reviews

The non‐intervention reviews had low reporting completeness and methodological quality, with an average reporting completeness of 51.8% for the MOOSE checklist and a compliance completeness of 50.5% for the DART. These reviews provided sufficient information on the background and results, but lacked details on the processes, such as manual search, selection, and data extraction. Few reviews have assessed or reported the quality of the included studies. Among these reviews, nearly 40% (33/83) used the Agency for Healthcare Research and Quality (AHRQ) tool to assess the quality of the primary studies. The AHRQ tool can address different healthcare questions, such as effectiveness, diagnosis, prognosis, and aetiology (Mamikutty et al., 2021). However, as previously stated, it also must be adapted to complex questions and social science settings. None of the reviews examined bias from confounding factors, outcome reporting bias, or loss to follow‐up.

#### 6.1.3 Summary of main results on the factors

We found that sources of literature, number of authors, publication year, and funding source declaration were significant predictors of reporting completeness and methodological quality in both intervention and non‐intervention systematic reviews in social science research. Intervention reviews published in journals indexed in the CSSCI had lower reporting completeness (46.7% vs. 51.1%) and methodological quality (39.6% vs. 47.9%) than those published in other journals, whereas non‐intervention reviews from journals indexed in the CSSCI had higher reporting completeness than those from other journals (53.3% vs. 50.3%). This may be because observational studies were more focused on exploring why the intervention works rather than on identifying what works (Davies, 2006; Snilstveit, 2012).

The number of authors was positively associated with reporting completeness and methodological quality of both intervention and non‐intervention reviews, meaning that more authors had higher quality than those with fewer authors, except for the non‐intervention reviews with a single author, which had the lowest overall quality. Systematic reviews require at least two researchers to perform tasks such as study selection and data extraction independently, thereby reducing the risk of errors (Cumpston & Flemyng, 2024).

The reporting completeness and methodological qualities of both intervention and non‐intervention systematic reviews in social science research improved over time, whereas the reporting completeness of abstracts in intervention reviews did not change. Researchers who have translated and explained reporting standards in China, such as the PRISMA statement and AMSTAR, have made significant contributions to this improvement (Ge et al., 2017; Moher et al., 2009; Tao et al., 2018; Tian et al., 2015; Xiong & Chen, 2011; Zhan, 2010; Zhang et al., 2015). To enhance the reporting quality of abstracts, the PRISMA working group updated the PRISMA statement that provides specific guidance for abstracts of systematic reviews (Page et al., 2021). Funding also influenced the quality of the intervention reviews, as funded reviews were of higher quality than unfunded reviews. This could be attributed to the funding enabling better research outputs, or the researchers seeking future funding (Thelwall et al., 2023).

### 6.2 Overall completeness and applicability of evidence

This methodological review covers systematic intervention and non‐intervention reviews of social science topics published in Chinese social science journals between January 2009 and December 2022. The review findings inform future systematic reviews in Chinese social science journals (and possibly in other disciplines) on how to improve methodological quality and report the completeness of policy reviews. However, this review excludes reviews with advanced synthesis methods, such as cross‐sectional meta‐analysis, cumulative meta‐analysis, Bayesian meta‐analysis, meta‐regression, meta‐analytic structural equation modelling, and network meta‐analysis; therefore, its applicability to these reviews is unclear.

### 6.3 Potential biases in the review process

We appraised the studies in pairs using two groups, each extracting data independently, to ensure reliable data extraction and quality assessment. To ensure inter‐group consistency, we randomly selected one of every 20 eligible reviews for extraction by all four reviewers and resolved any inter‐group disagreements through discussion. We did not evaluate the overall methodological quality of the intervention and non‐intervention reviews, as the AMSTAR‐2 tool does not provide weights for each item and the DART tool has high subjectivity and heterogeneity in rating the overall evidence confidence.

The methodological quality of non‐intervention reviews varies across research fields and may be influenced by publication volume in each field. For example, psychology, education, and management science account for approximately 90% of non‐intervention reviews in the social sciences. Furthermore, we assessed reporting completeness by combining partially and fully reported items in the factor analysis. However, some items may have more than two components and their relative importance may differ. Thus, our estimates may not fully reflect the reporting completeness in social science reviews in China.

### 6.4 Agreements and disagreements with other studies or reviews

Researchers from China have analyzed the quality of social science systematic reviews and meta‐analyses in 2021 (Bai et al., 2022). They randomly selected 200 reviews published in journals indexed in the CSSCI and used the PRISMA statement and AMSTAR to assess the reporting and methodological quality of both intervention and non‐intervention reviews, which may not be valid (Page et al., 2021; Shea et al., 2017). They found that both the reporting completeness and methodological quality of systematic reviews in China's social sciences were below the average level; 81.5% did not fully report the inclusion criteria, 42.5% did not fully report the search results, 89.5% did not fully report data extraction, 63.0% did not fully report the synthesis of results, and 73.4% did not fully report the risk of bias across the studies. They also found that the quality improved over time, which is consistent with our findings.

In 2021, Yu et al. developed the PRIOR‐MA (Preferred Reporting Items for Open and Reproducible Meta‐analysis) checklist for psychology research and assessed 68 meta‐analyses on psychology published in Chinese journal using PRIOR‐MA. The results indicated unsatisfactory overall reporting completeness of the included reviews: no reviews provided information on the registration and protocol, the search and selection process was rarely fully reported, the search time period was only disclosed in 8.7% of the reviews, the individual synthesis results were fully reported in 17.4%, and the risk of bias across studies was fully reported in 8.7% of reviews (Y. Liu et al., 2021).

The reporting completeness of abstracts is low in systematic intervention reviews in the social sciences (similar to non‐intervention reviews). Evidence assessment tools are seldom used in systematic reviews in the social sciences, either for intervention or non‐intervention reviews (31.3% vs. 11.5%, respectively). Similar findings were reported in other systematic reviews (Bai et al., 2022; Drucker et al., 2016; Lan et al., 2023; S. Li et al., 2014; T. Li et al., 2020; D. Liu et al., 2016; Wang et al., 2021).

## 7 AUTHOR'S CONCLUSIONS

### 7.1 General conclusions

We conducted a comprehensive and valuable assessment of the reporting completeness and methodological quality of systematic reviews across social sciences in China. Our research featured the following: (1) we included Chinese systematic reviews without restriction on the type of questions; reviews on effectiveness of the intervention, prevalence of the phenomenon, and correlational estimates were all included. (2) We included Chinese systematic reviews without restriction on the research fields; reviews on 19 fields in social science were included, such as Education, Management Science, Psychology. (3) We examined both the reporting completeness and methodological quality of intervention and non‐intervention reviews separately using PRISMA, AMSTAR‐2, MOOSE, and DART tool, and identified the common weaknesses and gaps in the review process, such as the lack of registration, protocol, risk of bias assessment, and data and code sharing. (4) We also explored the factors that may influence the reporting completeness and methodological quality of both intervention and non‐intervention reviews, such as the sources of literature, number of authors, publication year, and declaration of funding sources.

### 7.2 Implications and practice

Our study has important implications for both practice and social science research. It can help improve the quality and usability of systematic reviews and meta‐analyses by informing users of systematic reviews, such as policymakers, practitioners, and the public about the strengths and limitations of the existing evidence and the need for critical appraisal and interpretation of the results (Kolaski et al., 2023). It can also help guide the conduct and publication of systematic reviews and meta‐analyses by providing training and support for researchers and journals in the social sciences to use standards, tools, and platforms effectively.

We suggest that researchers and journals adopt and adapt reporting guidelines in healthcare to conduct and report systematic reviews and meta‐analyses and the specific characteristics and challenges of social science research (Bai et al., 2022). We also recommend that more attention be paid to the quality of non‐intervention reviews, which are prevalent in social sciences. Only few reviews cited the PRISMA guideline, language could be a barrier for non‐English researchers when following the reporting guidelines or checklists. International organization, such as EQUATOR network, could publish reporting guidelines in multiple language to promote their application in global research. Meanwhile, the authors should consider collaborating with more international teams to mitigate language bias and enhance the generalizability of their findings.

## CONTRIBUTIONS OF AUTHORS

The review was drafted by Guo LP; the literature retrieval was performed by Guo LP and Wei ZZ, and checked by Sarah M; the studies screening were performed by Ren JJ, Huang XY, and Xing X; the data extraction and quality assessment were conducted by Ren JJ, Huang XY, Guo LP, and Xing X; the data analysis was conducted by Guo LP and Zhou WJ; All the process were under the supervision of White H and Yang KH.

## DECLARATIONS OF INTEREST

Sarah Miller is the co‐chair of the Campbell Education Coordinating Group, Howard White is the head of Campbell Collaboration Research Programme.

## SOURCES OF SUPPORT

### 1 Internal sources

There is no source supported.

### 2 External sources

Liping Guo is supported by the China Scholarship Council.

Kehu Yang is supported by funding of the Major Project of the National Social Science Fund of China: Research on the Theoretical System, International Experience, and Chinese Path of Evidence‐based Social Science (No. 19ZDA142).

## Supporting information

Supporting information.

Supporting information.

Supporting information.

## Footnotes

1
https://www.cnki.net/

2
https://www.wanfangdata.com.cn/index.html

3
http://qikan.cqvip.com/

4
https://rayyan.qcri.org/

5
https://www.campbellcollaboration.org/

6
https://staff.amee.org/AMEE/AMEE/Publications/BEMELanding.aspx

7
https://osf.io/
